# Life Insurance

**Published:** 1880-12

**Authors:** 


### LIFE INSURANCE WITHIN THE REACH OF ALL.

Life insurance, which most people deem a duty, has hitherto been a luxury too costly for persons of moderate means. Life insurance companies, in order to accumulate their enormous surplus funds, amounting to many millions of dollars in many cases, have fixed their rates of premium at figures which, we repeat, are prohibitory. Every suggestion as to the propriety of reducing these rates has been met by vigorous outcry and opposition.

The extravagance of what we may properly call this old system of insurance has driven great numbers of persons to the adoption of the newer and better system of both life and accident insurance, which have been devised and introduced within the last few years, and which has brought insurance within the reach of those to whom it is of the greatest use, to wit, persons of moderate means.

The theory of all mutual life insurance is of course this: A great number of persons, presumably of respectability and in good health, are accepted as members. These persons contribute a yearly tax or premium which is payable in advance, and which is collected for the purpose of creating a fund out of which to pay the policies of such of the members as may die, paying the working expenses of the association, and creating a reasonable reserve fund. In effect, when a member dies his policy is paid by a contribution levied upon all the members. Instead of levying, however, the exact sum required for the payments above named, these companies levy a certain annual tax, calculated according to certain antiquated mortality tables, which annual sum must be paid in advance. It must be that the sums so collected are much more than are necessary for the payment of policies and expenses, for we have the spectacle of accumulated surplus funds amounting in one case to close upon a hundred millions of dollars, and in many cases to several millions. In other words, the history of nearly every one of the great life insurance companies shows that the policy holders or contributors are unduly taxed in order that gigantic and useless surplus funds may accrue, and the officers may be enriched by overgrown salaries and commissions.

Whenever any one of the many hundreds of the regular members of the New York Stock Exchange dies a tax which is trifling in amount is levied on each member, and $10,000 in ready money are at once paid to the family of the deceased member. There is no costly or useless machinery employed in this model insurance company, the little tax is cheerfully paid and the money is handed over, without ceremony or delay, to those who need it.

If trustworthy men could be found who would select a few thousand persons of respectability who are in good health, and would, without unnecessary expense, form them into a mutual company or association, on the same plan as that of the insurance department of the Stock Exchange, each member engaging to contribute only what might be needed to pay the policies of such of his fellow members as might die, and the reasonable expenses of the management, life insurance would cost but a tithe of the sum it is made to cost under the old system, and policy holders would be even more secure.

We have great satisfaction in informing our readers that insurance companies, upon the improved plan to which we have referred, do exist, and that they are eminently successful, as they deserve to be. At the suggestion of eminent men of business, who are known to us, we have carefully investigated the system and standing of two or three of these, and the result is that we have withdrawn from one of the old companies, and taken a policy in one of the really mutual ones. We are about to take a policy, also, in an accident insurance company which is conducted on the same excellent plan. The cost of our life insurance is less than a fourth the cost of a policy for the same amount in any of the old companies, and we believe the security to be at least as good. We shall be glad to furnish to any of our readers who are interested in life insurance (and all who have any one dependent upon them should feel an interest in it) the means of forming a correct judgment as to the wisdom of the course we have adopted. We have never had any hesitation in recommending any good thing to our readers after we have deliberately investigated and adopted it. We will forward all necessary printed information to any reader of the JOURNAL who will take the trouble to write to us on the subject.

What is true of life insurance is true also of accident insurance. Under the new and better system a policy of $4,500 against fatal accident, insuring also $25 per week during disability from accident, costs not over $8 per annum, or less than one-fourth the price charged by the old companies. No prudent man should hesitate to avail himself of this beneficent system.

---

FATHER : "Charley, I see no improvements in your marks." Charley : "Yes, papa, it is high time you had a serious talk with the teacher, or else he'll keep on that way forever."

